# The structure of DarB in complex with Rel^NTD^ reveals nonribosomal activation of Rel stringent factors

**DOI:** 10.1126/sciadv.ade4077

**Published:** 2023-01-18

**Authors:** Andres Ainelo, Julien Caballero-Montes, Ondřej Bulvas, Karin Ernits, Kyo Coppieters ‘t Wallant, Hiraku Takada, Sophie Z. Craig, Gabriel Mazzucchelli, Safia Zedek, Iva Pichová, Gemma C. Atkinson, Ariel Talavera, Chloe Martens, Vasili Hauryliuk, Abel Garcia-Pino

**Affiliations:** ^1^ Cellular and Molecular Microbiology, Faculté des Sciences, Université libre de Bruxelles 10 (ULB), Boulevard du Triomphe, Building BC (1C4 203), 1050 Brussels, Belgium.; ^2^ Institute of Organic Chemistry and Biochemistry, Academy of Sciences of the Czech Republic, v.v.i., Flemingovo nam. 2, 166 10 Prague 6, Czech Republic.; ^3^ Department of Biochemistry and Microbiology, University of Chemistry and Technology, Prague, Technicka 5, 166 28 Prague 6, Czech Republic.; ^4^ Department of Experimental Medical Science, Lund University, 221 00 Lund, Sweden.; ^5^ Centre for Structural Biology and Bioinformatics, Universite Libre de Bruxelles (ULB), Boulevard du Triomphe, Building BC, 1050 Bruxelles, Belgium.; ^6^ Faculty of Life Sciences, Kyoto Sangyo University, Kamigamo Motoyama, Kita-ku, Kyoto 603-8555, Japan.; ^7^ Mass Spectrometry Laboratory, MolSys Research Unit, Liège Université, B-4000 Liège, Belgium.; ^8^ University of Tartu, Institute of Technology, 50411 Tartu, Estonia.; ^9^ WELBIO, Avenue Hippocrate 75, 1200 Brussels, Belgium.

## Abstract

Rel stringent factors are bifunctional ribosome-associated enzymes that catalyze both synthesis and hydrolysis of the alarmones (p)ppGpp. Besides the allosteric control by starved ribosomes and (p)ppGpp, Rel is regulated by various protein factors depending on specific stress conditions, including the c-di-AMP–binding protein DarB. However, how these effector proteins control Rel remains unknown. We have determined the crystal structure of the DarB_2_:Rel^NTD^_2_ complex, uncovering that DarB directly engages the SYNTH domain of Rel to stimulate (p)ppGpp synthesis. This association with DarB promotes a SYNTH-primed conformation of the N-terminal domain region, markedly increasing the affinity of Rel for ATP while switching off the hydrolase activity of the enzyme. Binding to c-di-AMP rigidifies DarB, imposing an entropic penalty that precludes DarB-mediated control of Rel during normal growth. Our experiments provide the basis for understanding a previously unknown mechanism of allosteric regulation of Rel stringent factors independent of amino acid starvation.

## Teaser

The structure DarB in complex with RelNTD reveals how Rel stringent factors are activated independent of amino acid starvation.

## INTRODUCTION

The alarmones ppGpp and pppGpp, collectively referred to as (p)ppGpp, are ubiquitous nucleotide messengers that control bacterial growth, metabolism, antibiotic tolerance, and virulence (*1*–*3*). Both synthesis and degradation of (p)ppGpp are catalyzed by enzymes belonging to the RelA SpoT homolog (RSH) protein family (*4*). These can be divided into two classes: short RSHs and long RSHs. The latter group includes intricately regulated multidomain enzymes RelA, SpoT, and Rel (*4*–*6*). The stringent factor RelA is a monofunctional ribosome-associated (p)ppGpp synthetase activated during amino acid starvation (*7*). SpoT is a potent (p)ppGpp hydrolase with a weak synthesis activity (*8*, *9*) that became monofunctional in the Moraxellaceae lineage (*4*, *10*). While the phylogenetic distribution of RelA and SpoT is limited to Beta- and Gammaproteobacteria (*4*, *5*), the most taxonomically widespread long RSH representative, Rel, is a bifunctional ribosome-associated (p)ppGpp synthetase/hydrolase that, similarly to RelA, senses amino acid starvation on the ribosome (*11*, *12*). For both *Bacillus subtilis* Rel and *Escherichia coli* RelA, this activity is strongly stimulated by alarmone binding to a dedicated allosteric site (*13*–*15*).

Long RSH enzymes share a common domain architecture. The N-terminal domain region (NTD) of bifunctional Rel and SpoT is composed of two enzymatic domains: HD [catalyzes (p)ppGpp hydrolysis] and SYNTH [catalyzes (p)ppGpp synthesis] (*4*, *5*). An open-closed dynamic regulates the mutually exclusive HD and SYNTH activities of the NTD, with the open NTD state being SYNTH active (SYNTH^ON^ HD^OFF^) and closed being HD active (SYNTH^OFF^ HD^ON^) (*16*, *17*). This intra-NTD dynamics is exploited for regulation by (p)ppGpp in Rel: By binding in the pocket located at the interface between HD and SYNTH domains (formed by α9-α10 of HD and α11 of SYNTH), the alarmone promotes the open NTD state, thus stimulating (p)ppGpp production (*15*).

The regulatory C-terminal domain region (CTD) consists of four nonenzymatic domains: TGS, helical, ZFD, and RRM (*4*, *18*). Through extensive intramolecular contacts with the NTD via a core domain, the CTD controls the global conformational state of the enzyme to regulate its enzymatic output (*10*, *12*, *13*, *19*, *20*). Off the ribosome, HD-competent RSHs SpoT and Rel assume a compact state that suppresses the SYNTH activity of the NTD through cis-autoinhibition mechanism (*10*, *12*). In this state, the CTD domains TGS and helical directly stimulate hydrolysis by the HD domain, while ZFD and RRM suppress the SYNTH activity by precluding the guanosine diphosphate (GDP) and adenosine triphosphate (ATP) substrate binding (*10*, *12*). Upon association with starved ribosomes—i.e., stalled ribosomal elongation complexes harboring a cognate deacylated tRNA in the A site—Rel/RelA assumes a highly elongated SYNTH-active state in which the CTD is decoupled from the NTD (*18*, *21*–*23*). This conformation change ablates the CTD’s stimulatory effect on the HD, in effect precluding (p)ppGpp hydrolysis, while the SYNTH activity is strongly induced (*18*, *20*–*23*).

In addition to the well-studied regulation of long RSH enzymes by starved ribosomes, it has been shown that other bacterial adaptor proteins, such as the acyl carrier protein, YtfK, PtsN, and NirD, could interact with different long RSH domains to modulate the enzymatic output (*24*–*27*). However, the molecular bases of these regulatory mechanisms remain elusive. It was recently shown that, in *B. subtilis*, the small dimeric protein DarB binds directly to Rel (Rel*_Bs_*) NTD to induce (p)ppGpp synthesis while suppressing hydrolysis (*28*). Size exclusion chromatography revealed that dimeric DarB binds two molecules of Rel*_Bs_*, thus forming a DarB_2_:Rel_*Bs*2_ heterotetrameric complex (*28*). Tantalizingly, DarB-mediated control of Rel*_Bs_* is further regulated by cyclic di–adenosine monophosphate (c-di-AMP), a pleotropic messenger nucleotide (*29*). c-di-AMP binds to the cystathionine beta-synthase (CBS) domains of the DarB dimer (*28*) and abrogates DarB’s effect on Rel*_Bs_* (*28*). Furthermore, it was also shown that the DarB ortholog CbpB from *Listeria monocytogenes* also interacts with Rel to activate its SYNTH activity, and the stimulatory effect is similarly countered by c-di-AMP (*30*). The x-ray structure of *L. monocytogenes* CbpB/DarB revealed that apo- and c-di-AMP:CbpB are very similar (*30*), thus raising the question of how nucleotide binding abrogates the CbpB/DarB-mediated regulation of Rel.

Here, we use x-ray crystallography, hydrogen-deuterium exchange coupled to mass spectrometry (HDX-MS), isothermal titration calorimetry (ITC), fluorescence-based binding assays, and ppGpp synthesis and hydrolysis assays in a reconstituted stringent response system to (i) uncover the mechanistic basis of DarB-mediated regulation and (ii) dissect the interplay between Rel regulation by starved ribosomal complexes and DarB.

## RESULTS

### c-di-AMP suppresses DarB_2_ dynamics to preclude Rel binding

**Fig. 1.**
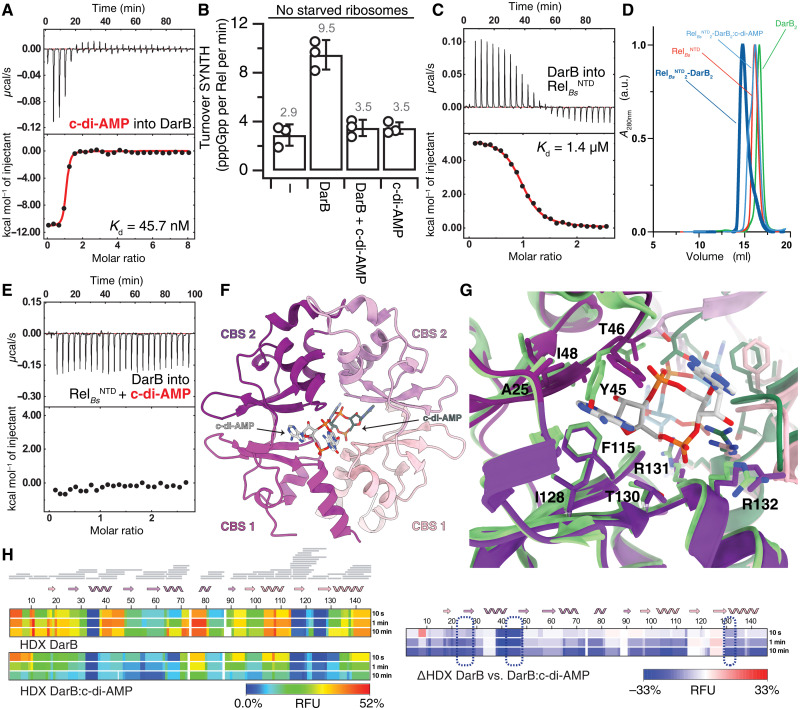
DarB:c-di-AMP conformational interplay. (**A**) Binding of c-di-AMP to DarB monitored by ITC. (**B**) Effect of c-di-AMP on DarB and its Rel-dependent activation of pppGpp synthesis. Rel*_Bs_* (250 nM) was incubated with ATP and GTP, in the absence or presence of saturating DarB (10-fold excess), c-di-AMP–saturated DarB, or c-di-AMP. (**C**) Binding of Rel to DarB monitored by ITC. (**D**) Analytical size exclusion chromatography of the Rel*_Bs_*^NTD^_2_-DarB_2_ complex (in dark blue), Rel^NTD^ (in red), DarB (in green), and the Rel*_Bs_*^NTD^_2_-DarB_2_ complex incubated with c-di-AMP (in light blue). The experiment confirms that the presence of c-di-AMP is sufficient to disrupt the complex. (**E**) Titration of DarB into c-di-AMP + Rel monitored by ITC. (**F**) Structure of DarB bound to c-di-AMP. Individual CBS domains of the tandem are labeled. Only one of the two c-di-AMP molecules is shown in the figure for clarity. (**G**) Structural details of the c-di-AMP–binding sites of DarB in the c-di-AMP–bound complex are shown in violet and of apo-DarB in green. Residues involved in the dinucleotide coordination are labeled. (**H**) Heatmaps representing the HDX of DarB (top) and DarB:c-di-AMP complex (center) and the ΔHDX (bottom). Residues involved in the binding to c-di-AMP are outlined by a dashed blue line. RFU, relative fractional uptake; a.u., arbitrary units; *A*_280nm_, absorbance at 280 nm.

As monitored by ITC, *B. subtilis* DarB has high affinity to c-di-AMP [dissociation constant (*K*_d_) of 45.7 nM] (Fig. 1A and table S1). The interaction is highly enthalpic (Δ*H* = −11.1 kcal/mol) and entropically unfavored (−*T*Δ*S* = 1.3 kcal/mol at 20°C), as expected from the strong electrostatics involved in the coordination of the phosphates from c-di-AMP. This indicates that DarB likely rigidifies upon binding c-di-AMP. In agreement with earlier reports (*28*, *30*), while the addition of unliganded DarB activates the SYNTH activity of full-length Rel*_Bs_*, the addition of DarB supplemented with 25 μM c-di-AMP does not (Fig. 1B).

Isolated NTD fragments of Rel enzymes (Rel^NTD^) are widely used to probe the mechanistic basis of Rel catalysis and its control, since they retain both the intramolecular SYNTH:HD regulation and stimulatory SYNTH control by (p)ppGpp (*15*–*17*, *23*, *31*). Because CbpB/DarB recognizes Rel via the NTD (*28*, *30*), we used *B. subtilis* Rel^NTD^ (Rel*_Bs_*^NTD^; residues 1 to 373; containing HD, SYNTH, and core domains) as a tool to study the Rel:DarB interaction. We measured the affinity of DarB to Rel*_Bs_*^NTD^ by ITC (Fig. 1C). Our results (*K*_d_ of 1.4 μM) are in agreement with earlier estimates by Krüger and colleagues (*28*) (*K*_d_ of 0.65 μM). c-di-AMP disassembles the DarB:Rel*_Bs_*^NTD^ complex (Fig. 1D), and binding of DarB to Rel*_Bs_*^NTD^ is completely abrogated in the presence of saturating c-di-AMP (Fig. 1E), thus explaining why the c-di-AMP–liganded DarB does not stimulate the SYNTH activity of full-length Rel*_Bs_*.

To characterize the interaction of c-di-AMP with DarB, we determined the structure of *B. subtilis* DarB bound to c-di-AMP to a resolution of 1.5 Å (Fig. 1E and table S2). c-di-AMP binds near the junction of the two tandem CBS domains of DarB, in the central channel of the donut-shaped DarB dimer (Fig. 1F). Both adenine bases project toward the exit of the channel, while the cyclic diphospho-ribose moieties are buried inside the channel. The hydroxyphenol group of Y45 intercalates between the adenine bases of each nucleotide in a stacking arrangement. One of the nucleotides interacts with a small hydrophobic pocket formed by residues I19, V24, A25, I48, P49, F115, I128, and T130, while the other is only tethered by Y45. The phosphates are coordinated by T46 and R131 and link the dimer via electrostatic interactions with R132 of the other subunit (Fig. 1G). The comparison between the unbound [Protein Data Bank (PDB) 6YJ8] and c-di-AMP–bound (this study) DarB reveals that the two structures are almost identical, with only minor deviations in the side-chain orientation of Y45. This result is in good agreement with earlier x-ray structures of *L. monocytogenes* CbpB/DarB—unliganded and complexed with c-di-AMP—which also revealed how c-di-AMP bridges the two CbpB/DarB monomers without inducing large-scale changes in the protein structure (*30*). Given the entropy-driven thermodynamics of c-di-AMP binding (Fig. 1A and table S1), we reasoned that, while not changing the overall structure of DarB, ligand binding could have a strong impact in protein dynamics.

To probe this conjecture, we monitored the overall dynamics of DarB bound to c-di-AMP using HDX-MS. This technique reports on solvent accessibility and H-bond stability of labile backbone amide protons (*32*). After deuterium labeling at various time points and subsequent quenching, the protein samples are enzymatically cleaved, and peptides are analyzed by MS for quantification of deuterium uptake. The difference in H/D exchange rates between conditions contains information about changes in conformation and local dynamics. We expressed the differences in deuterium uptake as changes in the relative fractional uptake (ΔRFU). In agreement with the entropically unfavored binding energetics as determined by ITC, the decrease in deuterium uptake throughout the protein in the presence of c-di-AMP indicates that binding to the dinucleotide traps DarB in a rigid state (Fig. 1H). Collectively, our results suggest that c-di-AMP counters the DarB-mediated control of Rel through abrogating the complex formation between the two proteins by imposing an entropic penalty on the interaction. To gain the necessary molecular detail, we next carried out structural studies of the DarB:Rel*_Bs_*^NTD^ complex.

### DarB stimulates (p)ppGpp synthesis by Rel*_Bs_* through direct interaction with the SYNTH domain

**Fig. 2.**
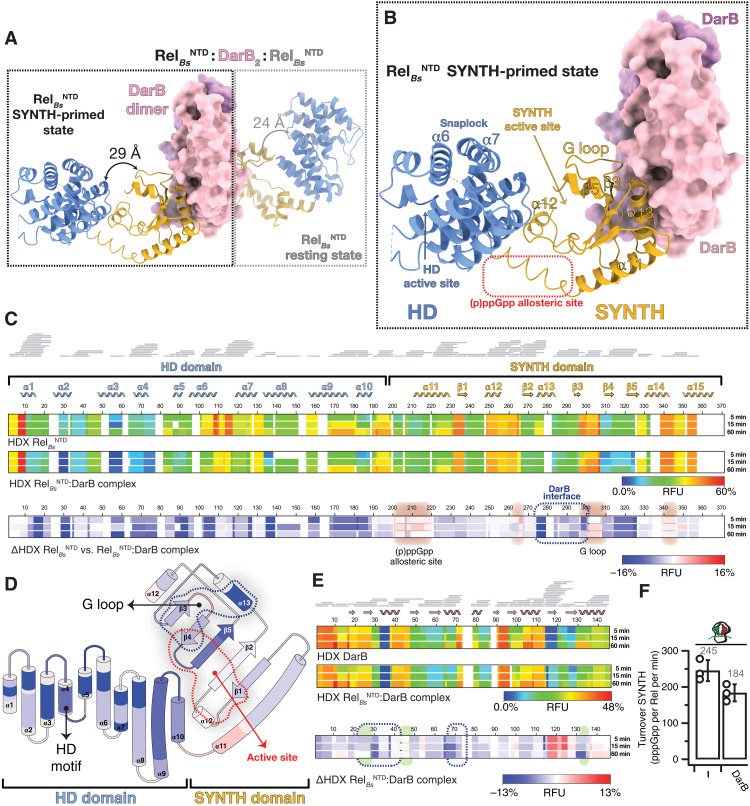
Structure of the Rel*_Bs_*^NTD^_2_:DarB heterotetrameric complex. (**A**) Crystal structure of DarB_2_:Rel*_Bs_*^NTD^_2_ heterotetrameric complex with the disc-shaped DarB dimer located at the center of the complex (colored in pink and lilac) and the two Rel*_Bs_*^NTD^ bound at both sides of the DarB dimer. For each Rel*_Bs_*^NTD^ molecule of the complex, the HD domain is colored in light blue, and the SYNTH domain is in yellow. In the nonsymmetrical hetero-complex, the Rel*_Bs_*^NTD^ in the SYNTH-primed state (left, outlined with a black dashed line) is observed in a more open and less structured conformation than the resting Rel*_Bs_*^NTD^ molecule (right, outlined with a light gray dashed line). The relative HD-to-SYNTH distance in each Rel*_Bs_*^NTD^ monomer is indicated. (**B**) Details of Rel*_Bs_*^NTD^ in the SYNTH-primed state highlighting key structural elements. (**C**) Heatmaps showing the HDX signal kinetics of Rel*_Bs_*^NTD^ (top) and Rel*_Bs_*^NTD^ as part of the DarB_2_:Rel*_Bs_*^NTD^_2_ complex (center) and the ΔHDX (bottom). Both catalytic domain of Rel*_Bs_* and all the secondary structural elements of the NTD are shown in the figure. Residues involved in the binding interface with DarB are outlined by a dashed blue line, and the regions with increased deuterium uptake, which include the G loop that becomes exposed upon binding to DarB and the alarmone allosteric site, are shaded in red. (**D**) Topology representation of Rel*_Bs_*^NTD^ colored as a function of the ΔHDX. (**E**) Heatmaps representing the HDX of DarB (top) and DarB as part of the DarB_2_:Rel*_Bs_*^NTD^_2_ complex (center) and the ΔHDX (bottom). Residues involved in the binding interface with Rel*_Bs_*^NTD^ are indicated by a dashed blue line, and those involved in the binding to c-di-AMP are shaded in green. (**F**) Effect of DarB on the SYNTH activity of Rel*_Bs_* in the presence or absence of “starved” ribosomes.

To gain a high-resolution structural insight into DarB-mediated regulation of Rel, we determined the x-ray structure of DarB_2_:Rel^NTD^_2_ at a resolution of 2.9 Å (Fig. 2, A and B). In agreement with the heterotetrameric architecture predicted by size exclusion chromatography analysis (*28*), our structure reveals a DarB dimer engaging two Rel*_Bs_*^NTD^ monomers, with Rel*_Bs_*^NTD^ polypeptides not forming direct contacts with each other. The two tandem CBS domains of DarB bridge the complex, with CBS1 and CBS2 interacting with the SYNTH domain of different Rel*_Bs_*^NTD^ molecules (Fig. 2A).

The heterotetramer does not have internal twofold symmetry, and the two Rel*_Bs_*^NTD^ molecules adopt slightly different conformations induced by different lattice contacts, a feature observed in other long RSH homologs and proteins that explore multiple conformations (fig. S1A) (*16*, *33*). While one Rel*_Bs_*^NTD^ assumes a more compact state reminiscent of the resting (SYNTH^OFF^ HD^OFF^) state of the enzyme observed in the structure of *Thermus thermophilus* Rel*_Tt_*^NTD^ (*17*) [root mean square deviation (RMSD) of 0.96 Å], the other Rel*_Bs_*^NTD^ attains a more open state (Fig. 2B), resembling that of the SYNTH-active (SYNTH^ON^ HD^OFF^) Rel*_Tt_*^NTD^ bound to ppGpp and AMP (*17*) (RMSD of 0.91 Å) (fig. S1, B and C). This observation suggests that the increase in synthetase activity caused by DarB binding is due to shifting the conformational ensemble toward the open state of the enzyme, de facto precluding the active hydrolase state.

To capture the effect of DarB binding on the dynamics of the complex, we turned to HDX-MS. By comparing the deuterium exchange of DarB:Rel*_Bs_*^NTD^ with that of isolated Rel*_Bs_*^NTD^, we observed increased protection from the exchange of the entire hydrolase domain, which indicates an overall rigidification of the HD domain upon DarB binding. In agreement with the crystal structure of the complex, a strong ΔHDX signal is clustered in the region of the SYNTH domain that encompasses helices β3 and α13 (residues I270 to N306) of Rel*_Bs_*^NTD^, confirming that this region directly contacts DarB. Notably, while binding of DarB leads to an overall protection of the Rel enzyme, two functionally important regions are comparatively more dynamic: (i) residues L206 to V218 that are located in allosteric (p)ppGpp-binding site (*15*) and (ii) residues A252 to R266 of the SYNTH active site that are involved in GDP/guanosine triphosphate (GTP) substrate coordination (Fig. 2, C and D). This is in agreement with the observed movement and partial unwinding of α11, the hinge connecting both catalytic domains, and consistent with allosteric stimulation of SYNTH activity by DarB.

The structure of the DarB_2_:Rel*_Bs_*^NTD^_2_ complex complemented by the HDX data provides a mechanistic explanation for the stimulation of Rel’s SYNTH activity while repressing HD activity. By promoting the formation of the open SYNTH^ON^ HD^OFF^ state of Rel*_Bs_*, the two crucial HD active site elements—the α6/α7 snaplock motif and the HD loop (*17*)—are partially disordered, and their positions are incompatible with (p)ppGpp binding and hydrolysis. This is coupled to the SYNTH active site residues of the SYNTH^ON^ HD^OFF^ Rel*_Bs_*^NTD^ molecule observed in a more dynamic state than in the case of the second, resting-state Rel*_Bs_*^NTD^ molecule in the Rel*_Bs_*^NTD^_2_:DarB_2_ complex.

### DarB-mediated control of Rel SYNTH is independent of the ribosomal stringent response

While DarB associates with Rel with relatively high affinity (micromolar range), the effective affinity of Rel to starved ribosomes is significantly higher, in the submicromolar range (*13*). Comparison of the structure of *B. subtilis* Rel in the complex with starved ribosomes (*23*) with that of Rel_2_:DarB_2_ suggests that ribosomal recruitment is incompatible with DarB-mediated control of Rel enzymatic activities.

We used our reconstituted in vitro *B. subtilis* stringent response system (*34*) to assay the effect of DarB on the SYNTH activity of full-length Rel*_Bs_* activated by *B. subtilis* starved ribosomal complexes (i.e., 70*S* initiation complexes supplemented with A site–cognate deacylated *E. coli* tRNA^Val^). As expected, while DarB stimulates the SYNTH activity when tested with isolated Rel*_Bs_* (Fig. 1B), DarB has a mild inhibitory effect on Rel-mediated ^3^H-pppGpp synthesis when the stringent factor is activated by starved ribosomes (Fig. 2F), likely due to the competition of DarB with the ribosome for Rel binding.

Given that the allosteric binding of (p)ppGpp to Rel is one of the hallmarks of the stringent response in *B. subtilis* (*15*), we measured by ITC the affinity of the Rel*_Bs_*^NTD^-DarB complex for pppGpp (*K*_d_ = 8.2 μM; fig. S1D), which was similar to that of Rel*_Bs_*^NTD^ for pppGpp (*K*_d_ = 10.6 μM) (*13*). While the affinities remained comparable, the binding thermodynamics changed (*13*). In the presence of DarB, the interaction of the alarmone with Rel becomes threefold more enthalpic and slightly entropically unfavorable (table S1), indicating that the alarmone allosteric site is exposed in the Rel*_Bs_*^NTD^-DarB complex. These results are also consistent with the rigidification of Rel upon complex formation observed by HDX-MS and suggest that DarB restricts the conformational space of Rel. Collectively, these results suggest that (i) SYNTH stimulation by DarB and starved ribosomes is not synergetic, (ii) the mechanism of activation by DarB is independent of the amino acid starvation pathway and triggers structural rearrangement that is equivalent with the opening of the SYNTH domain induced by pppGpp, and (iii) in the context of amino acid starvation, regulation by starved ribosomes would override that by DarB.

### DarB engages the Rel*_Bs_*^NTD^ SYNTH domain in the vicinity of the GDP binding site

Our x-ray structure reveals that DarB engages Rel*_Bs_*^NTD^ through structural elements involved in the coordination of the GDP substrate in the SYNTH active site: β3, α13, and the N-terminal region of the so-called G-loop (Fig. 2D) (*35*). From the DarB side, HDX-MS validated the structure of the complex (Fig. 2E), confirming that the binding interface on DarB involved β1, β2, α1, and α2. When bound to Rel*_Bs_*^NTD^, the β7-β8 region of DarB (which is part of c-di-AMP–binding site) exhibits strong deuterium uptake compared to apo-DarB. The increased dynamics upon binding in this part suggest that it is allosterically linked to the Rel*_Bs_*^NTD^ binding interface, indicating a possible pathway controlling the opposing effects of c-di-AMP versus Rel*_Bs_*.

**Fig. 3.**
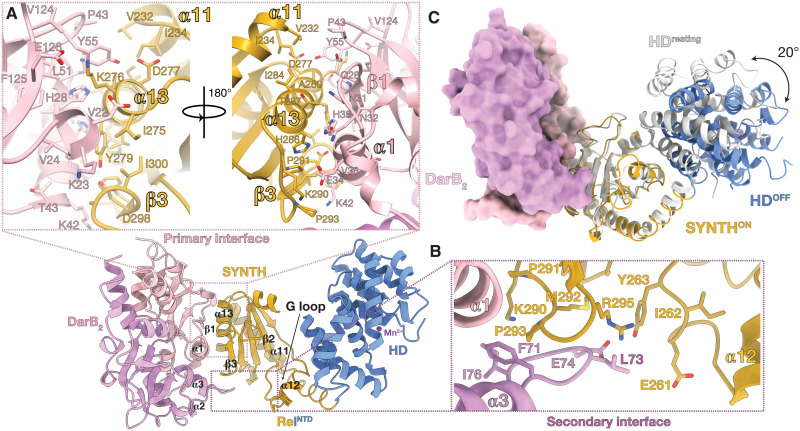
DarB interacts with Rel*_Bs_* via the SYNTH domain. (**A**) Rel*_Bs_*:DarB primary interaction interface involving α13 and β3 from Rel*_Bs_* and α1, α2, β1, and β2 from DarB. (**B**) Rel*_Bs_*:DarB secondary interaction interface formed between the α13/β3 connecting loop from Rel*_Bs_* adjacent to the SYNTH active site and the α2/α3 loop of DarB. (**C**) Superposition of Rel*_Bs_*^NTD^ in the SYNTH-primed state (colored as per Fig. 2C) onto Rel*_Bs_*^NTD^ in the resting state shown in light gray (PDB ID 6YXA). The 20° movement of the HD domain away from the SYNTH domain observed in Rel*_Bs_*^NTD^ in complex with DarB compared with the resting Rel*_Bs_* (PDB ID 6YXA) is indicated with a black arrow.

The primary interface of the complex occupies 822.8 Å^2^ (Fig. 3A) and is formed mainly by α1 (N32 to T43) and β1 (A25 to Q28) of the CBS2 domain of DarB that complements hydrophobic patches of Rel*_Bs_*^NTD^ located in α13 (I275 to T287) and β3 (F296 to Y298). The linker region between CBS1 and CBS2 provides additional contacts to the N-cap of α13 (Fig. 3A). A small secondary 230.5-Å interface is formed between the CBS1 domain of the other DarB subunit and the highly conserved β3α13 ^291^PXPGR^295^ loop, effectively bridging the complex (Fig. 3B). DarB residues G72 to I76 of this additional anchor point contribute contacts via the α2/α3 loop of DarB, which provide further stabilization to the SYNTH active site through the SYNTH α13/β3 loop. All these interactions likely contribute to increased dynamics and loss of structure in the SYNTH active site, particularly at the region involving Rel*_Bs_*^NTD^ α12, which expands to α11 and results in the unwinding of its N-terminal part (R193 to K209) and the ≈30° movement of the HD domain away from SYNTH, compared to its position in the resting state of the enzyme (Fig. 2A). Overall, these structural observations are consistent with the higher deuterium uptake observed in α13 upon binding to DarB (Figs. 2D and 3C).

**Fig. 4.**
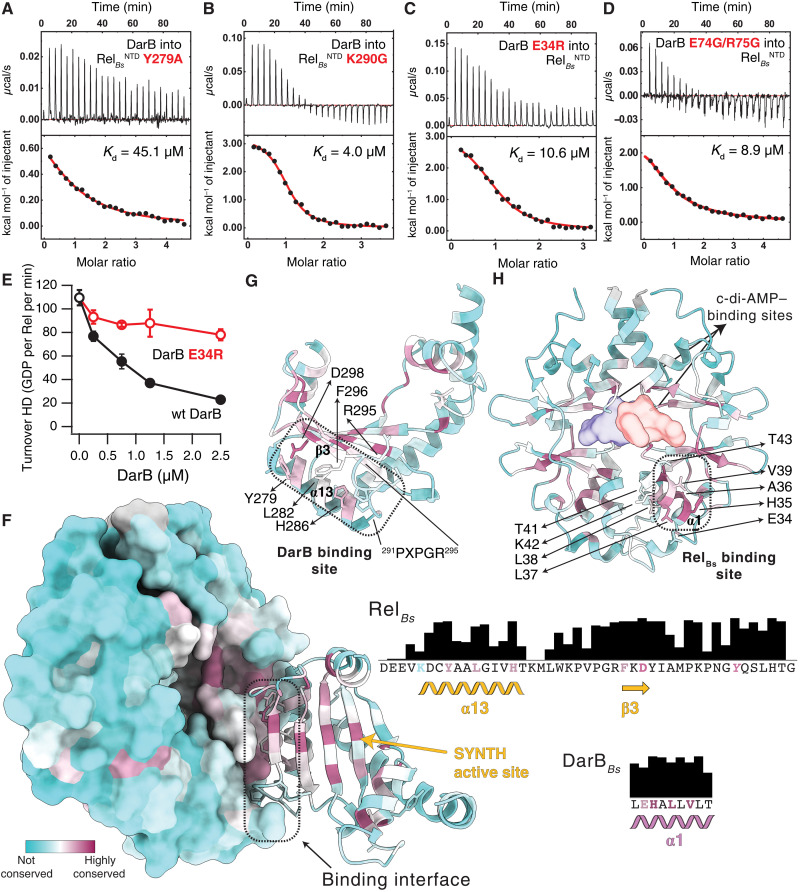
Biophysical and biochemical interrogation of the Rel*_Bs_*:DarB binding interface. Effect of the Y279A (**A**) and K290G (**B**) substitutions in Rel*_Bs_*^NTD^ to the interaction with DarB, monitored by ITC. Effect of the substitutions to the primary E34R (**C**) or secondary E74G/R75G (**D**) DarB interfaces, to the binding to Rel*_Bs_*^NTD^, monitored by ITC. (**E**) Effect of an E34R substitution on DarB to the activity of DarB monitored as a function of the hydrolase activity of Rel*_Bs_*. (**F**) DarB-SYNTH interface color-coded by the conservation score of each amino acid calculated by ConSurf. Residues involved in the primary interface are shown in the conservation bar plots to the right and colored on the basis of their individual conservation profile. The strictly conserved Y of the G loop of Rel is shown in italic. Structural elements of SYNTH (**G**) and DarB (**H**) colored as in (F) underline the strong conservation of the binding interface. The contact regions between both proteins are outlined by dashed black lines with the residues directly involved in the primary binding interface and the PXPGR motif highlighted in (G) and (H) and the location of the c-di-AMP shown as a surface in (H).

Next, we probed the interfaces revealed by the crystal structure through mutagenesis. Using ITC, we characterized complex formation between substituted Rel*_Bs_*^NTD^ and DarB variants. In agreement with strong deuterium protection observed in the region involving I269 to A280, substitution Y279A (completely buried in the interface) decreased the affinity of Rel*_Bs_*^NTD^ to DarB 32-fold, whereas the K290G substitution, which is located slightly off this region, results in a mere 3-fold decrease in affinity (Fig. 4, A and B, and table S1). On the DarB side, the substitution of E34R led to a 7.5-fold decrease in affinity of DarB^E34R^ for Rel*_Bs_*^NTD^ (Fig. 4C and table S1). In addition, the substitutions E74G/R75G (Fig. 3B) to the DarB residues that contribute to the secondary interface decreased the affinity for Rel*_Bs_*^NTD^ by 6.4-fold (Fig. 4D and table S1). This suggests that this interface not only supports the main binding site but also contributes significantly to the interaction.

As was shown earlier, DarB suppresses the HD activity of Rel*_Bs_* (*28*). In good agreement with Krüger and colleagues (*28*), saturation of Rel*_Bs_* with DarB leads to a 5.5-fold drop in the rate of ^3^H-ppGpp hydrolysis (Fig. 4E). By contrast, saturating amounts of DarB^E34R^ (10-fold excess compared to the concentration of the enzyme) triggered only a 1.4-fold drop in hydrolysis (Fig. 4E).

### DarB-mediated regulation of Rel is likely widespread

The CBS domain that constitutes DarB is widespread across the tree of life. It is found as a standalone domain or as part of a variety of proteins always occurring as pairs, forming the αββα Bateman module [see fig. S1E (*36*) and data S1 for a detailed phylogenetic tree of CBS domains, including in green the sequences that were used in the conservation analysis] and involved in the binding of adenosine nucleotides. The DarB subfamily is mainly limited to Gram-positive bacteria, with no homologs detected in *Staphylococcus aureus* (*37*). Similarly, RSH enzymes are also broadly distributed and encoded in the core genome of most bacterial species with bifunctional Rels absent in Beta- and Gammaproteobacteria (*4*). Therefore, we used ConSurf to probe the evolutionary conservation of the DarB-Rel interface and mapped the residue conservation on the surface of the two proteins (Fig. 4, F to H, and data S2 and S3, listing the sequence ID and annotation of Rel and DarB, respectively).

The DarB:Rel interface and the G(T)DP and ATP binding sites are the most conserved regions in the Rel SYNTH domain (Fig. 4G). Residues of that domain form the primary DarB:Rel interface—including Y279, which is essential for Rel binding to DarB—and are more than 80% conserved. In the secondary DarB:Rel interface, the ^291^PXPGR^295^ motif connecting α13 and β3 is 70% conserved, while the overall Rel protein sequence conservation in our set is below 35% sequence identity. On the DarB side, the residues of α1 that are involved in the coordination of Rel, but not in nucleotide binding or DarB dimerization, are more than 90% conserved (Fig. 4H). This conservation complementarity becomes even more apparent when we compare the conservation pattern of bacterial CBS α1 versus their eukaryotic homologs (fig. S2A), which have completely diverged and do not interact with Rel (not present in metazoans). A similar observation can be made between monofunctional and bifunctional long RSH synthetases. Dedicated (p)ppGpp synthetases such as RelA, which are mainly under ribosome control and unlikely to interact with DarB homologs, have a very different conservation pattern in the β3 region that connects directly with DarB α1 (fig. S2B), which overlaps with a region of SYNTH involved in G nucleotide substrate specificity (*38*) and the N-cap of α13, which interacts with the RRM domain of Rel in the HD^ON^ τ state (but not in RelA) (*10*). Thus, it appears that DarB has evolved to recognize a conserved multifunctional Rel hotspot rather than the interfaces having coevolved as such. Collectively, the conservation patterns suggest that DarB-mediated control of Rel activity is likely widespread in Gram-positive bacteria.

### Loss of protein dynamics induced by c-di-AMP precludes DarB from binding to Rel*_Bs_*

The incorporation of c-di-AMP prevents the interaction of DarB with Rel*_Bs_*^NTD^ and the associated increase in (p)ppGpp production (Fig. 1, B and D). HDX-MS shows that c-di-AMP triggers a strong rigidification of DarB, including loss of dynamics in the regions that are part of the binding interface with Rel*_Bs_* (Fig. 1G). Conversely, in contrast with the more rigid c-di-AMP–bound state of DarB, the Rel*_Bs_*^NTD^-bound state of DarB is characterized by increased dynamics in the dinucleotide binding pocket located at the DarB dimer interface (Fig. 1G).

In addition to the effects on protein dynamics revealed by HDX, comparison of x-ray structures of *B. subtilis* DarB bound to c-di-AMP with Rel*_Bs_*^NTD^-bound DarB reveals only minor differences. These differences were mainly restricted to the adenine recognition site. Crucially, in the presence of the dinucleotide, the C-terminal cap of DarB’s α1 helix moves ≈6° away from the core of the protein, projecting out K42, while Y45 changes conformation to intercalate the c-di-AMP (fig. S3, A and B). These changes likely lead to clashes with F296 and K297 of Rel*_Bs_* (fig. S3B), which would preclude complex formation between the two proteins. In addition, when we compared the quaternary arrangement of the dimers, we observed a rotation of ≈4° of the twofold symmetry axis in the DarB–c-di-AMP complex likely triggered by the proximity of the phosphates from both dinucleotides in the center of the dimer (fig. S3C). This rearrangement abolishes the internal symmetry of the DarB dimer and results in important changes in the regions V27 to T46 and the α2/α3 loop, both involved in Rel*_Bs_* recognition that precludes binding to Rel*_Bs_*.

Last, our structural results are consistent with the suppressor mutants in *L. monocytogenes* hypothesized to compromise Cbp/DarB:Rel complex formation. Substitutions H35Y, L38H, D68V, and G72R in *L. monocytogenes* Cbp (residues H35, L38, N68, and G72 in *B. subtilis* DarB) and in the conserved ^278^CYA^280^ sequence motif (C278F and A280T) of *L. monocytogenes* Rel (also ^278^CYA^280^ in Rel*_Bs_*) are found in the complex interface (fig. S4A) (*30*). These nonconservative substitutions likely compromise the stability of the complex with Rel. By contrast, D68 (N68 in *B. subtilis* DarB), while not directly part of the complex interface, is involved in the stabilization of the DarB dimer by interacting with α1 of the neighboring subunit (fig. S4B). Thus, the D68V phenotype reinforces the important role of the local orientation of both DarB monomers in the function of DarB.

### DarB specifically stimulates binding of the ATP SYNTH substrate to Rel

The stimulatory effect of pppGpp on the *E. coli* long RSH RelA (RelA*_Ec_*) is mediated by a marked increase in affinity for ATP to the SYNTH domain (*15*). We hypothesized that DarB-mediated stimulation of the (p)ppGpp synthesis by Rel could also use a similar pathway. The close proximity of the interaction interface of DarB to Rel’s G-loop as well as DarB-induced structural changes in both SYNTH active site and allosteric (p)ppGpp-binding sites all speak in favor of this possibility.

**Fig. 5.**
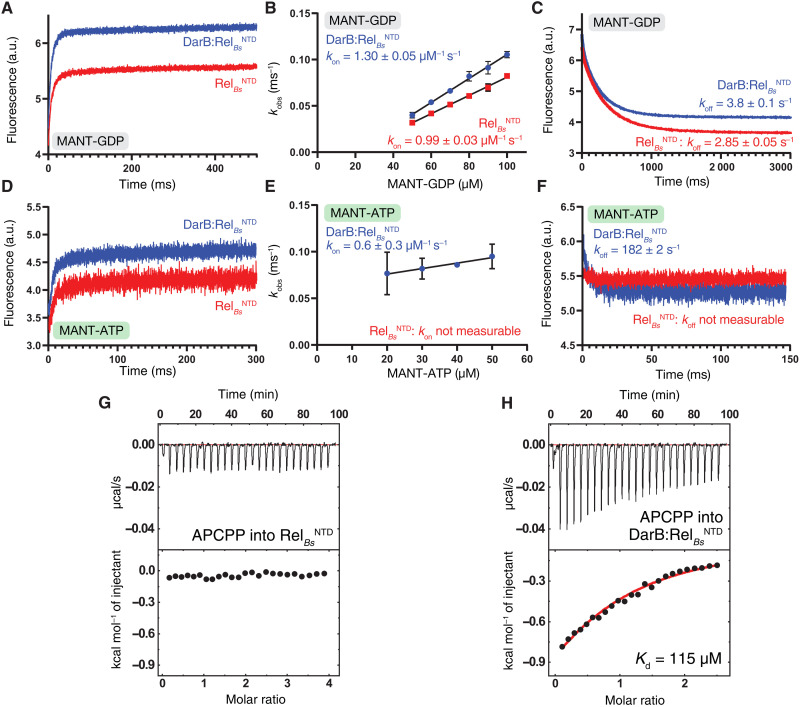
Kinetics and thermodynamics of nucleotide binding to Rel*_Bs_* in the presence and absence of DarB. (**A**) Kinetics of MANT-GDP binding to Rel*_Bs_*^NTD^ (in red) and DarB:Rel*_Bs_*^NTD^ (in blue) monitored by stopped flow. The interaction was measured by FRET excitation of MANT fluorescence upon mixing 10 μM protein with increasing concentrations of MANT-GDP (**B**). (**C**) Kinetics of MANT-GDP dissociation from Rel*_Bs_*^NTD^ (in red) and DarB:Rel*_Bs_*^NTD^ (in blue). (**D**) Kinetics of MANT-ATP binding to Rel*_Bs_*^NTD^ (in red) and DarB:Rel*_Bs_*^NTD^ (in blue). The interaction was measured as in (A) by FRET excitation of MANT-ATP fluorescence upon mixing 10 μM protein with increasing concentrations of MANT-ATP (**E**). (**F**) Kinetics of MANT-ATP dissociation from Rel*_Bs_*^NTD^ (in red) and DarB:Rel*_Bs_*^NTD^ (in blue). In both cases, the dissociation was monitored upon rapid mixing with an excess (2 mM) of unlabeled GDP or ATP. Binding of APCPP to Rel*_Bs_*^NTD^ (**G**) and DarB:Rel*_Bs_*^NTD^ (**H**) monitored by ITC.

We assayed the effects of DarB on the binding kinetics of GDP and ATP to Rel*_Bs_*^NTD^ using fluorescent nucleotide derivatives 2′/3′*N*-methylanthraniloyl GDP (MANT-GDP) and MANT-ATP. In the case of MANT-GDP, both association (*k*_on_) and dissociation (*k*_off_) rates—and therefore the calculated equilibrium affinity (*K*_d_^MANT-GDP^ = *k*_off_/*k*_on_)—are not affected by DarB (Fig. 5, A to C). By contrast, the addition of DarB markedly alters the binding kinetics of MANT-ATP (Fig. 5, D to F). While, in the case of Rel*_Bs_* assayed in the absence of DarB, we could not reliably quantify the MANT-ATP binding due to the slow association and fast dissociation rates, markedly increased *k*_on_ and decreased *k*_off_ rates in the presence of DarB allowed a reliable equilibrium *K*_d_^MANT-ATP^ affinity estimate of 308 μM. We complemented our kinetic experiments measuring the binding thermodynamics of the nonhydrolyzable ATP analog adenosine-5′-[(α,β)-methyleno]triphosphate (APCPP) to Rel*_Bs_*^NTD^ by ITC. While in the absence of DarB [similarly to RelA*_Ec_*^NTD^ in the absence of pppGpp (*15*)], Rel*_Bs_*^NTD^ alone has low affinity to APCPP (Fig. 5G), the affinity markedly increases in the presence of DarB (Fig. 5H). Notably, the affinity estimate of DarB:Rel*_Bs_* for APCPP obtained by ITC (*K*_d_^APCPP^ = 115 μM) is in the order of that obtained using MANT-ATP.

## DISCUSSION

**Fig. 6.**
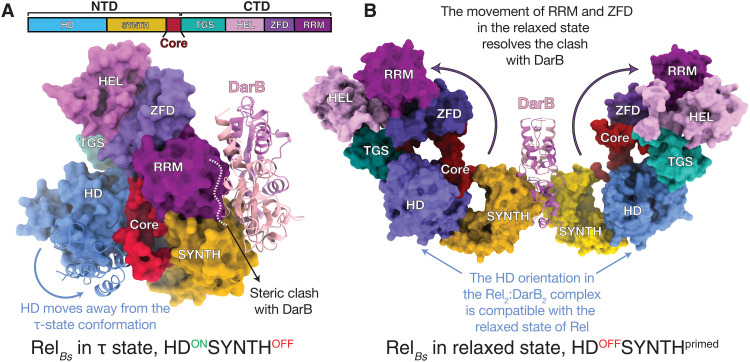
DarB as a conformational selector of Rel*_Bs_*. (**A**) Surface representation of an idealized (unrealistic) model of full-length Rel*_Bs_* in the hydrolase-compatible τ state superimposed on the crystal structure of the DarB:Rel*_Bs_*^NTD^ complex (used only to illustrate why access to the τ state is sterically blocked). Comparison of the two structures shows that the closed HD-active τ state is not compatible with the binding of DarB due to the sterical clash of DarB with the RRM and ZFD domains and the closing of the SYNTH active site by HD. (**B**) Model of the full-length Rel*_Bs_*:DarB heterotetrameric complex in the HD^OFF^ SYNTH^primed^ relaxed state. The rearrangement of RRM and ZFD domains allows for binding of DarB that, in turn, precludes the recoil of the CTD and corresponding inactivation of the HD. In both (A) and (B), Rel models were based on the structures of *A. baumannii* SpoT in the τ and relaxed states (*10*).

Our study establishes the mechanistic basis for DarB-mediated regulation of *B. subtilis* stringent factor Rel. DarB exploits the intrinsic functional dynamics of Rel to modulate its enzymatic output, tilting the conformational equilibrium and stabilizing a conformation that is compatible with alarmone synthesis but not hydrolysis. Specifically, DarB exploits the NTD open-closed dynamics that is similarly controlled by HD and SYNTH nucleotide substrates (*16*, *17*) and (p)ppGpp (*15*). The recent structure of *Acinetobacter baumannii* SpoT in an active hydrolase state suggests that efficient alarmone hydrolysis is only favored when the enzyme is in a compact τ-shaped state (*10*). A model of the DarB_2_:Rel_*Bs*2_ complex with the full-length Rel*_Bs_* in an active hydrolase τ state (*10*) indicates that the interaction of DarB with α13 of the full-length Rel*_Bs_* would sterically clash with the RRM domain, compromising the crucial interactions with SYNTH that maintain the compact HD^ON^SYNTH^OFF^ τ state (Fig. 6A). However, this interaction would be allowed in the less compact, hydrolase-incompatible (HD^OFF^SYNTH^primed^), relaxed state of the enzyme (Fig. 6B). The relaxed state (*10*) would be able to accommodate the HD domain moving away from SYNTH, resulting from the interaction with DarB. This is consistent with the significant drop in hydrolysis by Rel*_Bs_* upon binding to DarB.

The DarB-mediated regulation of Rel is structurally incompatible with a recently described structure of a dimer of a partially C-terminally truncated *B. subtilis* Rel that lacks the RRM and ZFD domains (*23*). In this dimer, the DarB-binding interface of C-terminally truncated Rel is completely buried as part of the dimer interface (fig. S5). However, given the relatively low propensity of Rel for dimerization [*K*_d_ of 10.6 μM (*23*); no dimerization is detectable for 50 nM Rel (*13*)] as compared to Rel’s affinity to DarB [*K*_d_ estimates ranging from 0.65 (*28*) to 1.4 μM (this work)], it is unlikely that Rel dimerization would play a role in DarB-mediated regulation in the cell. CBS domains respond to ligand binding with conformational changes of variable magnitude. The small changes in conformation observed in DarB upon binding to c-di-AMP are typical of standalone regulatory CBS tandems (*36*). This behavior is likely due to the short length of the linker connecting the domains and an important feature that contributes to the ON/OFF nature of the DarB switch.

From a mechanistic viewpoint, DarB-mediated stimulation of Rel’s SYNTH activity is thus associated not only with an increased affinity to the ATP substrate, which is analogous to how (p)ppGpp promotes the SYNTH activity of RelA (*15*), but also with the interference with the hydrolase-enhancing effect of the CTD via destabilization of the τ state. This suggests that this could be a common regulatory pathway exploited by other NTD-targeting macromolecular allosteric regulators of long RSHs besides DarB, such as the recently found *E. coli* SpoT regulator YtfK (*26*) and the *E. coli* RelA regulator NirD (*25*), and also by regulators of Rel that target the CTD of the enzyme such as EIIA^NTR^, which inhibits (p)ppGpp hydrolysis in *Caulobacter crescentus* (*24*). In this context, the correlation of strong conservation of the allosteric interface of the complex and the allosteric regulation by alarmones suggests an evolutionary pressure to retain this mode of regulation that may be in place across bacteria.

It is interesting to reflect on how, given our affinity estimates, the DarB-mediated Rel regulation would work in the cell. DarB is approximately 10-fold less abundant (0.9 μM) (*39*) than ribosomes (10 μM) (*40*). This suggests that, under amino acid starvation, activation by ribosomes (which have higher affinity for Rel) would efficiently override the DarB-mediated regulation of Rel. The intracellular concentration of c-di-AMP in *B. subtilis* is estimated to range from 1 to 5 μM, with c-di-AMP levels increasing upon sporulation (*41*) and as intracellular K^+^ increases (*42*); c-di-AMP levels are reported to increase from 0.5 to 19 μM (*43*) for other bacterial species. In this context, DarB binds c-di-AMP with an affinity of 45 nM, ≈30-fold stronger than its affinity for Rel, so the formation of a stable DarB-Rel complex may be conditioned to other factors besides the fluctuations of the cytosolic levels of c-di-AMP. Therefore, stress conditions triggering the DarB-mediated activation of Rel must be coupled to the degradation of the dinucleotide or the binding of c-di-AMP to other stress effector molecules that could reduce the local concentration releasing DarB. It is thus clear that this conserved pathway involved in stress responses is still not complete; further integrative data must be gathered to address this question in a broader context.

## MATERIALS AND METHODS

### Construction of plasmids

All strains and plasmids used in this study are listed in table S3. For the pET24d-His10-SUMO-darB expression plasmid, the entire coding region of the *B. subtilis darB* gene was amplified by polymerase chain reaction (PCR) using synthetic oligonucleotides VHT923 (5′-ATCGCGAACAGATTGGTGGTATAAGCTTACAATCAGATCAACTTCTT-3′) and VHT924 (5′-AGTGCGGCCGCAAGCTTCACTTATTCAATGAGCGTATATGCTTATTC-3′) and *B. subtilis* genomic DNA as a template. To construct the desired plasmid VHP731 (pET24d-*His10-SUMO-darB*), Gibson assembly was performed to introduce the resulting PCR fragment into pET24d-*His10-SUMO* plasmid backbone, which was PCR-amplified from pET24d-*His10-SUMO-rel* (VHP186) using the synthetic oligonucleotides VHT852 (5′-ACCACCAATCTGTTCGCGATGAGCTTCAATGATGT) and VHT920 (5′-AGCTTGCGGCCGCACTCGAGC).

For the pET24d-His10-SUMO-darB and rel mutant expression plasmids, the entire plasmid was amplified by PCR using diverging oligonucleotides carrying the desired substitutions. The respective wild-type expression plasmids were used as templates. After PCR, the mixtures were treated with Dpn I to remove the template plasmid and subsequently ligated to yield the mutant expression plasmids.

For the Rel*Bs*^NTD^ Y279A substitution, oligonucleotides F-bsRel_Y279A (GCGGCGGTGCTTGGCATCATTCACACATGC) and R-bsRel_Y279A (GCAGTCCTTTATGCTATTCACAAGAATACGG) were used. For the Rel*Bs*^NTD^ K290G substitution, oligonucleotides F-bsRel_K290G (GGCCCGATGCCAGGCAGATTCAAAGATTATATCGC) and R-bsRel_K290G (CCAGCATGTGTGAATGATGCCAAGC) were used.

For the DarB E34R substitution, oligonucleotides F-darB_E34R (CGTCATGCATTATTAGTATTGAC) and R-darB_E34R (AAGGTTATTTCCGACTTGCACG) were used. For the DarB E74G/R75G double substitution, oligonucleotides darB_E74_Rev (AAGTCCAAAAATACTGTTCATGATC) and darB_E74GR75G_Fw (GGAGGCATTGAGTTTGAAAAGCTTGACCAAA) were used.

### Protein expression and purification for biochemical assays

For *B. subtilis* DarB, overexpression of DarB was performed in 80 ml of autoinduction media (*44*) supplemented with kanamycin (100 μg/ml) using VHP731 (for expression of wild-type DarB) or VHP1224 (for expression of E34R-substituted DarB). The culture was inoculated by a single colony from freshly transformed *E. coli* BL21 DE3 cells and grown for 18 hours at 30°C while shaking. The cells were harvested by centrifugation (7000*g* for 5 min), washed once by 20 ml of binding buffer [BB; 200 mM NaCl, 10% glycerol, 10 mM imidazole, 4 mM β-mercaptoethanol, and 25 mM Hepes:KOH (pH 7.5)], and resuspended in 20 ml of BB supplemented with 1 mM phenylmethylsulfonyl fluoride and deoxyribonuclease I (DNase I; 1 U/ml). Cells were lysed by one passage through a high-pressure cell disrupter (150 MPa, cooled to 4°C), cell debris was removed by centrifugation (25,000 rpm for 40 min; JA-25.50, Beckman Coulter rotor), and clarified lysate was taken for protein purification. Clarified cell lysate was filtered through a 0.2-μm syringe filter and loaded onto the HisTrap 5-ml HP column preequilibrated in BB. The column was washed with 6 column volume (CV) of BB, and the protein was eluted with a gradient of elution buffer [EB; 200 mM NaCl, 10% glycerol, 500 mM imidazole, 4 mM β-mercaptoethanol, and 25 mM Hepes:KOH (pH 7.5)]. Fractions most enriched in DarB (≈90 to 100% of EB) were pooled, totaling approximately 4 ml. The sample was then loaded on a HiPrep 10/26 desalting column preequilibrated with storage buffer [SB; 200 mM KCl, 10% glycerol, 4 mM β-mercaptoethanol, and 25 mM Hepes:KOH (pH 7.5)]. The fractions containing DarB were collected. To cleave off the His_10_-SUMO tag, 40 μl of His_6_-Ulp1 (2 mg/ml; Protein Expertise Platform facility at Umeå University) was added to the sample, and the mixture was incubated at room temperature for 30 min while gently rocking. To remove the cleaved-off His_10_-SUMO tag, the protein sample was passed through a second HisTrap 5-ml HP preequilibrated with SB. Fractions containing DarB were collected and concentrated on an Amicon Ultra centrifugal filter device with a cutoff of 10 kDa to a concentration of 1.2 mg/ml (70 μM). Protein preparation was aliquoted, frozen in liquid nitrogen, and stored at −80°C. The purity of protein preparations was assessed by SDS–polyacrylamide gel electrophoresis (PAGE).

### Protein production and purification for stopped flow assays

*E. coli* BL21 (DE3) cultures carrying pET24d plasmids for expressing either His10-SUMO-Rel_Bs_^NTD^ or His10-SUMO-DarB were grown overnight in LB medium containing kanamycin (50 μg/ml). The precultures were diluted 100-fold in 2× 1 liter of LB medium containing kanamycin (50 μg/ml) and 0.1% glucose and grown at 37°C. At an optical density at 600 nm of ≈0.6, isopropyl-β-d-thiogalactopyranoside at 0.5 mM was added, and the temperature was lowered to 28°C for overnight expression.

Expression cultures were harvested by centrifugation and resuspended in 12 ml of resuspension buffer [25 mM Hepes (pH 8.0), 200 mM KCl, 200 mM NaCl, 2 mM MgCl_2_, 1 mM tris(2-carboxyethyl)phosphine (TCEP), 0.002% mellitic acid, and 1 cOmplete EDTA-free Protease Inhibitor Cocktail tablet per liter]. DNase (10 μg/ml) was added to the suspension, and the cells were lysed by passage through an Emulsiflex-C3 homogenizer (Avestin). Lysates were cleared by centrifugation at 30,000*g* for 45 min at 4°C and vacuum-filtered through a 0.45-μm membrane filter.

The cleared lysates were loaded on a gravity-flow Co^2+^-affinity resin column equilibrated with purification buffer [25 mM Hepes (pH 8), 200 mM KCl, 200 mM NaCl, 2 mM MgCl_2_, 1 mM TCEP, and 0.002% mellitic acid]. The column was washed with 4 ml of purification buffer, followed by 4 ml of purification buffer containing 20 mM imidazole. The proteins were eluted by 4 ml of purification buffer containing 400 mM imidazole.

The imidazole concentration was reduced 100-fold by repeated concentration in centrifugal filtering units (30-kDa cutoff for Rel_*Bs*_^NTD^ and 10-kDa cutoff for DarB) and dilution with purification buffer. At a final volume of 10 ml, the His10-SUMO tag was cleaved by adding ≈1:100 Ulp1 protease and incubating overnight at 10°C.

The cleaved proteins were purified by passing the solution through a gravity-flow Co^2+^-affinity resin column and chasing with 2 ml of purification buffer. Last, the proteins were concentrated to 1 ml and run through a Superdex 200 (Rel*_Bs_*^NTD^) or 75 (DarB) 10/300 size exclusion chromatography column. The purity of the preparations was assessed by SDS-PAGE and pooled. To form the Rel_*Bs*_^NTD^:DarB complex, both proteins were concentrated to ≈500 μM and mixed together at a ratio of 3:1 to 4:1 of DarB to Rel*_Bs_*^NTD^. The complex was purified on a Superdex 200 10/300 size exclusion chromatography column with peak fractions assessed by SDS-PAGE and pooled. For *B. subtilis* Rel (Rel*_Bs_*), Rel*_Bs_* was expressed and purified as described previously in the work of Takada *et al*. (*34*).

### Enzymatic assays

Both *B. subtilis* Rel synthase and hydrolase activity assays were performed in Hepes:polymix buffer [20 mM Hepes:KOH (pH 7.5), 2 mM dithiothreitol, 5 mM Mg(OAc)_2_, 95 mM KCl, 5 mM NH_4_Cl, 0.5 mM CaCl_2_, 8 mM putrescine, and 1 mM spermidine] at 37°C as described earlier (*34*), with minor modifications. Specifically, hydrolase activity assay was performed in the presence of 100 μM ^3^H-ppGpp, 200 μM ppGpp, 1 mM MnCl_2_, and 0.25 μM *B. subtilis* Rel. The activity was measured alone or in the presence of 0.25 to 2.5 μM *B. subtilis* DarB, either wild type or E34R-substituted variant (1 to 10× molar excess over Rel). In all cases, DarB was first incubated in Hepes:polymix buffer with/without c-di-AMP for 5 min at 37°C before adding to the reaction mixture. Synthase activity assay was performed in the presence of 300 μM ^3^H-GTP, 700 μM GTP, 1 mM ATP, and 0.25 μM *B. subtilis* Rel with the addition of 2.5 μM DarB and/or 25 μM c-di-AMP.

For the experiment with the starved ribosomal complexes as described earlier (*34*), 2 μM *E. coli* deacylated tRNA^Val^ (Chemical Block Ltd.), 0.25 μM initiation complexes, and 10 μM ppGpp were added to the reaction mixture. After preincubation at 37°C for 3 min, the reaction was started by the addition of prewarmed Rel (for hydrolase assay) or ^3^H-GTP (for synthase assay). Five-microliter aliquots of the reaction mixture were taken throughout the time course of the reaction and quenched with 4 μl of 70% formic acid supplemented with a cold nucleotide standard (4 mM GDP/GTP) for ultraviolet (UV) shadowing. Individual quenched samples were spotted on PEI (Polyethylenimine) Cellulose plates for thin-layer chromatography (TLC) plates (Macherey-Nagel), and nucleotides were resolved in either 1.5 M KH_2_PO_4_ (pH 3.5) buffer (optimized for resolving pppGpp) or 0.5 M KH_2_PO_4_ (pH 3.5) (optimized for resolving ppGpp). The TLC plates were dried and cut into equally sized sections as guided by UV shadowing, and ^3^H radioactivity was quantified by scintillation counting in EcoLite Liquid Scintillation Cocktail (MP Biomedicals).

### Isothermal titration calorimetry

For all ITC measurements, Rel*_Bs_*^NTD^ and DarB samples were prepared as described above. In the case of the binding between pppGpp and the Rel*_Bs_*^NTD^_2_:DarB_2_ complex, the sample was prepared under the conditions described previously in the work of Van Nerom *et al*. (*45*). All titrations were performed with an Affinity ITC (TA Instruments) at 25°C. For the titration, DarB and DarB variants were loaded in the instrument syringe at 200 μM, and Rel*_Bs_*^NTD^ and the Rel*_Bs_*^NTD^ variants were used in the cell at 15 μM. The titrations were performed in 50 mM Hepes (pH 7.5), 500 mM KCl, 500 mM, NaCl, 10 mM MgCl_2_, 1 mM TCEP, and 0.002% mellitic acid. Final concentrations were verified by absorption using a NanoDrop One (Thermo Fisher Scientific). All ITC measurements were performed by titrating 2 μl of the sample in the syringe into the cell using a constant stirring rate of 75 rpm. All data were processed, buffer-corrected, and analyzed using the NanoAnalyze and Origin software packages. Thermodynamic parameters are shown in table S1.

### Crystallization and structure determination

Crystals of DarB_2_ in complex c-di-AMP grew in condition C4 of the ProPlex crystallization screen. The screening was carried out using the sitting-drop vapor diffusion method, and drops were set up in Swiss (MRC) 96-well two-drop UVP sitting-drop plates using the Mosquito HTS system (TTP Labtech). Drops of 0.1 μl of protein and 0.1 μl of precipitant solution were equilibrated to 80 μl of precipitant solution in the reservoir. The crystals were harvested using 20% glycerol as a cryoprotecting agent and vitrified in liquid N_2_ for transport and storage before x-ray exposure. X-ray diffraction data were collected at the SOLEIL Synchrotron (Gif-sur-Yvette, Paris, France) on the Proxima 1 (PX1) and Proxima 2A (PX2A) beamlines using an EIGER-X 16M detector.

The screening of crystallization conditions of the DarB_2_:Rel_*Bs*2_ complex was carried out using the sitting-drop vapor diffusion method. The drops were set up in Swiss (MRC) 96-well two-drop UVP sitting-drop plates using the Mosquito HTS system (TTP Labtech). Drops of 0.1 μl of protein and 0.1 μl of precipitant solution were equilibrated to 80 μl of precipitant solution in the reservoir. Commercially available screens were used to screen for crystallization conditions. The condition resulting in protein crystals (ProPlex screen position B5) was repeated as 2-μl drops. Crystals were harvested using suitable cryoprotecting solutions and vitrified in liquid N_2_ for transport and storage before x-ray exposure. X-ray diffraction data were collected at the SOLEIL Synchrotron (Gif-sur-Yvette, Paris, France) on the PX1 and PX2A beamlines using an EIGER-X 16M detector. Because of the high anisotropic nature of the data from all the crystals, we performed anisotropic cutoff and correction of the merged intensity data as implemented on the STARANISO server (http://staraniso.globalphasing.org/) using the DEBYE and STARANISO programs. The analysis of the data suggested a resolution of 2.97 Å (with 2.97 Å in a*, 3.18 Å in b*, 3.02 Å in c*, and 2.84 Å). The crystals of DarB_2_:c-di-AMP complex grew in 0.2 M lithium sulfate and 0.1 M MES 6.0 with 20% (w/v) polyethylene glycol 4000 and diffracted on average to ≈1.5 Å, while those of the DarB_2_:Rel_*Bs*2_ complex diffracted to ≈3.0 Å. All the data were processed with the X-ray Detector Software (XDS) suite (*46*) and scaled with Aimless. In all cases, the unit cell content was estimated with the program MATTHEW COEF from the CCP4 program suite (*47*). Molecular replacement (MR) was performed with Phaser (*48*).

For structure determination, as described in (*49*), we used the coordinates of Rel*_Tt_*^NTD^ as search model for the HD and SYNTH domains (PDB ID 6S2T) (*17*) and PDB ID 6YJ8 for DarB. The Molecular replacement (MR) solution from Phaser was used in combination with Rosetta as implemented in the MR-Rosetta (*50*) suit from the Phenix package (*51*). After several iterations of manual building with Coot (*52*) and maximum likelihood refinement as implemented in Buster (*53*) and phenix.refine from the Phenix package (*51*), the models were completed to *R*/*R*_free_ of 17.4/19.4% in the case of DarB_2_:c-di-AMP and *R*/*R*_free_ of 26.4/31.9% for the DarB_2_:Rel*_Bs_*^NTD^_2_ complex. Table S2 details all the x-ray data collection and refinement statistics.

### Hydrogen-deuterium exchange mass spectrometry

HDX-MS experiments were performed on an HDX platform composed of a Synapt G2-Si mass spectrometer (Waters Corporation) connected to a nanoACQUITY ultraperformance liquid chromatography (UPLC) system, as described in (*49*). Samples of Rel*_Bs_*^NTD^, DarB, and the Rel*_Bs_*^NTD^:DarB complex were prepared at a concentration of 20 to 50 μM. For each experiment, 5 μl of sample was incubated for 5, 15, or 60 min in 95 μl of labeling buffer L [50 mM Hepes, 500 mM KCl, 500 mM NaCl, 2 mM MgCl_2_, 1 mM TCEP, and 0.002% mellitic acid (pH 7.5)] at 20°C. The nondeuterated reference points were prepared by replacing buffer L by equilibration buffer E [50 mM Hepes, 500 mM KCl, 500 mM NaCl, 2 mM MgCl_2_, 1 mM TCEP, and 0.002% mellitic acid (pH 7.5)]. After labeling, the samples are quenched by mixing with 100 μl of prechilled quench buffer Q [1.2% formic acid (pH 2.4)]. Seventy microliters of the quenched samples was directly transferred to the Enzymate BEH Pepsin Column (Waters Corporation) at 200 μl/min and at 20°C with a pressure of 8.5 kpsi. Peptic peptides were trapped for 3 min on an ACQUITY UPLC BEH C18 VanGuard precolumn (Waters Corporation) at a flow rate of 200 μl/min in water [0.1% formic acid in HPLC water (pH 2.5)] before being eluted to an ACQUITY UPLC BEH C18 column for chromatographic separation. Separation was performed with a linear gradient buffer (7 to 40% gradient of 0.1% formic acid in acetonitrile) at a flow rate of 40 μl/min. Identification of peptides and deuteration uptake analysis was performed on the Synapt G2Si in (ESI+) - HDMS^E^ mode (Waters Corporation). Leucine enkephalin was applied for mass accuracy correction, and sodium iodide was used as calibration for the mass spectrometer. HDMS^E^ data were collected by a 20- to 30-V transfer collision energy ramp. The pepsin column was washed between injections using pepsin wash buffer [1.5 M Gu-HCl, 4% (v/v) methanol, and 0.8% (v/v) formic acid]. A blank run was performed between each sample to prevent significant peptide carryover. Optimized peptide identification and peptide coverage for all samples were performed from undeuterated controls (five replicates). All deuterium time points were performed in triplicate.

### Nucleotide binding kinetics measurements

Nucleotide binding kinetics to apo-Rel*_Bs_*^NTD^ and DarB:Rel*_Bs_*^NTD^ were measured using a μSFM stopped flow instrument connected to a MOS-500 spectrometer (BioLogic), following procedures previously described (*54*). In all cases, binding was detected by excitation at 280 nm and fluorescence detection through a 400-nm long-pass filter (Thorlabs). The observed signal increase upon nucleotide binding is due to fluorescence resonance energy transfer (FRET) from tryptophan residues in the enzyme to MANT-labeled GDP or ATP (Jena Bioscience) (*54*). Temperature was kept at 4°C by a circulating water bath during all measurements. To measure association rates, 20 μM enzyme was mixed 1:1 with a 5- to 10-fold molar excess of MANT-labeled nucleotide, and fluorescence change was observed over 200 to 500 ms. For ATP binding measurements, 3′dGDP was present in the mixes at a 10-fold molar excess over the enzyme. The measured binding curves were fit to single exponential functions, and observed rate constants were plotted against nucleotide concentration to obtain the association rate constant. To measure dissociation rates, mixtures of 20 μM enzyme, 100 to 160 μM MANT-labeled nucleotide, and, in MANT-ATP measurements, 200 μM 3′dGDP were prepared and mixed 1:1 with the corresponding unlabeled nucleotide in a 50-fold molar excess over the labeled nucleotide. All measurements were performed in 50 mM Hepes (pH 7.5), 500 mM KCl, 500 mM, NaCl, 10 mM MgCl_2_, 1 mM TCEP, and 0.002% mellitic acid. Fluorescence change was observed over 150 ms (MANT-ATP) or 3 s (MANT-GDP). The measured curves were fit to single exponential functions to directly obtain the dissociation rate constants. *K*_d_ values were calculated as the ratio of *k*_off_ and *k*_on_. Each curve was averaged from three to five technical repeats to reduce noise and independently measured three times. Values in the figures are means ± SD of three independent measurements.

### Conservation analysis

For the conservation analysis, we performed ConSurf runs (https://consurf.tau.ac.il/) (*55*) in standard mode, retrieving 150 sequences for DarB and 75 sequences of long RSH enzymes. For this, we used the PDB ID 6yj9 as entry for DarB and the coordinates of our own structure of Rel. In the resulting sets of sequences, all the long RSHs correspond to Rel homologs (leaving out Beta- and Gammaproteobacteria’s RelA and SpoT) and the DarB set correspondingly excluding CBS domain from Beta- and Gammaproteobacteria. The program performed multiple sequence alignments with MAFFT (Multiple Alignment based on the Fast Fourier Transform) from sequences retrieved from UniProt90 using HMMER as a homolog search algorithm (*E* value: 0.0001) and a criteria of maximal ID of 95% and minimal ID of 35%. ConSurf used phylogenetic trees calculated with neighbor joining and maximum likelihood (ML) distance. The accession numbers of ConSurf-identified sequences are listed in the Supplementary Materials. Additional sequence alignments of homologs of Rel and DarB were generated with MAFFT v7.490 with the L-INS-i strategy (*56*).

### Analytical size exclusion chromatography

For the analytical size exclusion chromatography, 0.5 mg of total protein in 250 μl (corresponding approximately to 45 μM Rel*_Bs_*^NTD^, 120 μM DarB, or 15 μM Rel*_Bs_*^NTD^_2_:DarB_2_ heterotetramer) was loaded onto a Superdex 200 10/300 Increase column at a flow of 1 ml/min. To assess the effect of c-di-AMP on the Rel*_Bs_*^NTD^_2_:DarB_2_ complex, 500 μM c-di-AMP (Jena Bioscience) was added to the sample, and the sample was immediately loaded onto the column.

## Supplementary Materials

### This PDF file includes:

Figs. S1 to S5 Tables S1 to S3 PDB validation files Data S1

## Notes

View/request a protocol for this paper from *Bio-protocol*.
