# Supplementary material for: The structure of DarB in complex with RelNTD reveals nonribosomal activation of Rel stringent factors
Source: Sci Adv. 2023 Jan 18;9(3):eade4077. doi: 10.1126/sciadv.ade4077 (PMC9848473; doi:10.1126/sciadv.ade4077)
Supplement: Supplementary file 1 — Figs. S1 to S5 Tables S1 to S3 PDB validation files Data S1 [file sciadv.ade4077_sm.pdf]

Science Advances
AAAS

Supplementary Materials for

The structure of DarB in complex with Rel^{NTD} reveals nonribosomal activation of Rel stringent factors

Andres Ainelo *et al.*

Corresponding author: Vasili Hauryliuk, vasili.hauryliuk@med.lu.se; Abel Garcia-Pino, abel.garcia.pino@ulb.be

Sci. Adv. **9**, eade4077 (2023)
DOI: 10.1126/sciadv.ade4077

The PDF file includes:

Figs. S1 to S5
Tables S1 to S3
PDB validation files
Data S1
Legends for data S2 and S3

Other Supplementary Material for this manuscript includes the following:

Data S2 and S3

Supplementary Figures

Fig. S1.

Structural features of *B. subtilis* Rel*Bs*^{NTD} in the DarB$_2$Rel$_{Bs}^{NTD}_2$ complex. (**a**) Analysis of the packing of the asymmetric unit content (red and blue Rel$_{Bs}^{NTD}$; pink and yellow DarB) and symmetry-related partners (in green) of the DarB$_2$Rel$_{Bs}^{NTD}$$_2$ complex. The Rel$_{Bs}^{NTD}$ monomer in the resting state (in blue) is highlighted by a blue dashed line and the Rel$_{Bs}^{NTD}$ monomer in the open state (in dark red, more loosely packed) is highlighted by a dark red dashed line. Comparison of the structure of Rel*Bs*^{NTD} in the DarB$_2$Rel$_{Bs}^{NTD}$$_2$ complex with *T. thermophilus* Rel$_{Tt}^{NTD}$. (**b**) SYNTH-primed (PDB ID 6S2U) and (**c**) resting (PDB ID 6S2V) states of Rel$_{Tt}^{NTD}$. (**d**) Binding of pppGpp to the Rel$_{Bs}^{NTD}$$_2$:DarB$_2$ complex monitored by ITC. (**e**) Topological representation of DarB with the individual CBS domains coloured in pink and dark violet and the nucleotide binding site outlined by a dashed black line. The *αββα* Bateman modules that define each CBS domain are shaded in light blue and pink.

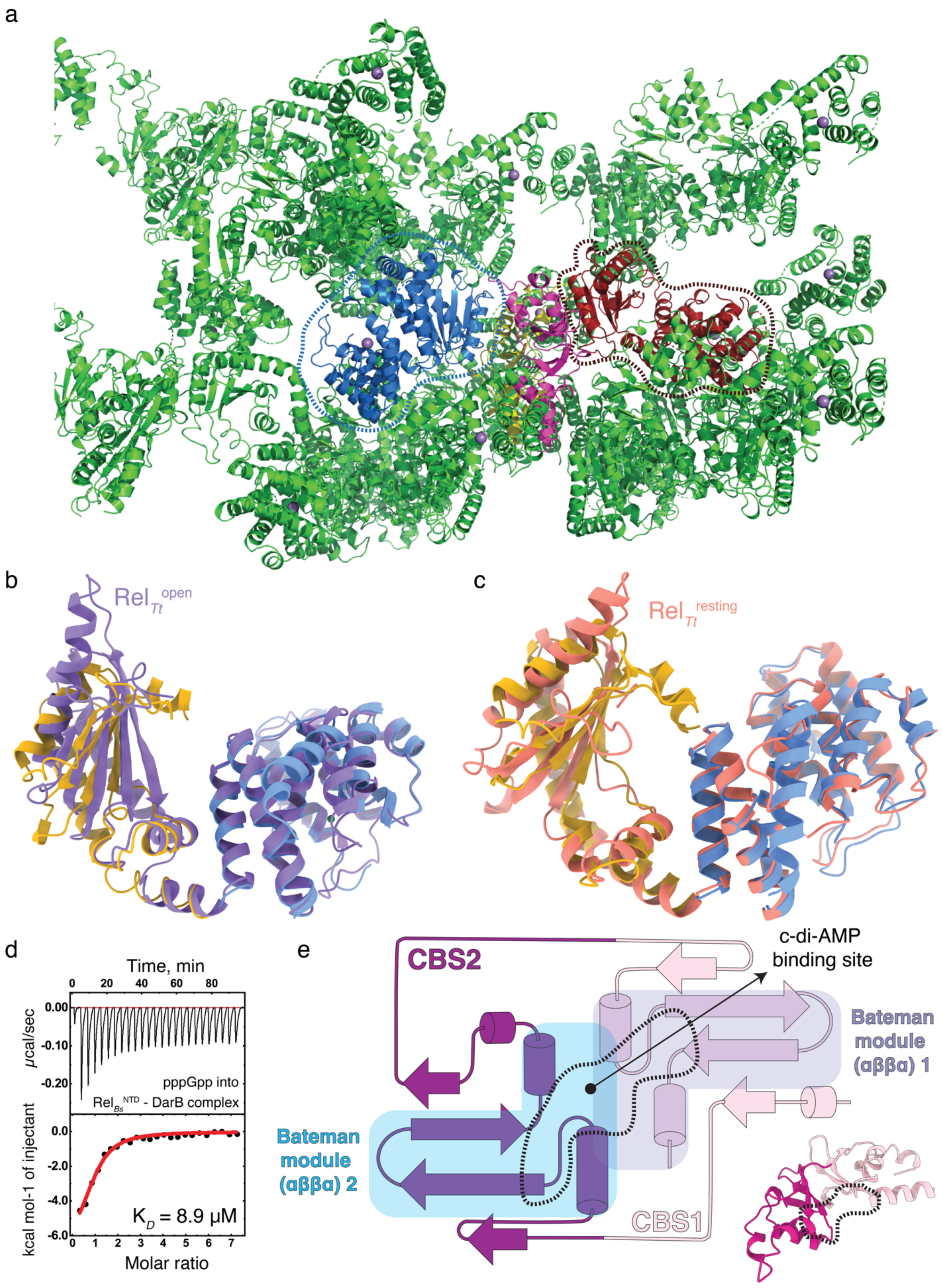
a
b
RelTt open
c
RelTt resting
d
Time, min
0 20 40 60 80
µcal/sec
0.00
-0.10
-0.20
pppGpp into
RelBs NTD - DarB complex
kcal mol-1 of injectant
0.0
-2.0
-4.0
-6.0
0 1 2 3 4 5 6 7
KD = 8.9 µM
Molar ratio
e
CBS2
c-di-AMP
binding site
Bateman
module
(αββα) 1
Bateman
module
(αββα) 2
CBS1

Fig. S2.

Conservation of the amino acid involved in the complex formation between DarB and Rel. (**a**) Representative DarB sequences are aligned with homologous eukaryotic CBS domains, the red dots are marking the Rel - DarB interface while red shadings highlight the c-di-AMP – DarB interface. (**b**) Sequence alignment of representatives Rel, RelA and SpoT long RSH enzymes. The SYNTH domain region is highlighted by a thick black line and the sequences corresponding to monofunctional RelA enzymes are boxed in black. The ^{278}CYA^{280} interface motif of *L. monocytogenes* Rel is shown with (*) and the sequences of α13 and β3 are highlighted with a blue shadow.

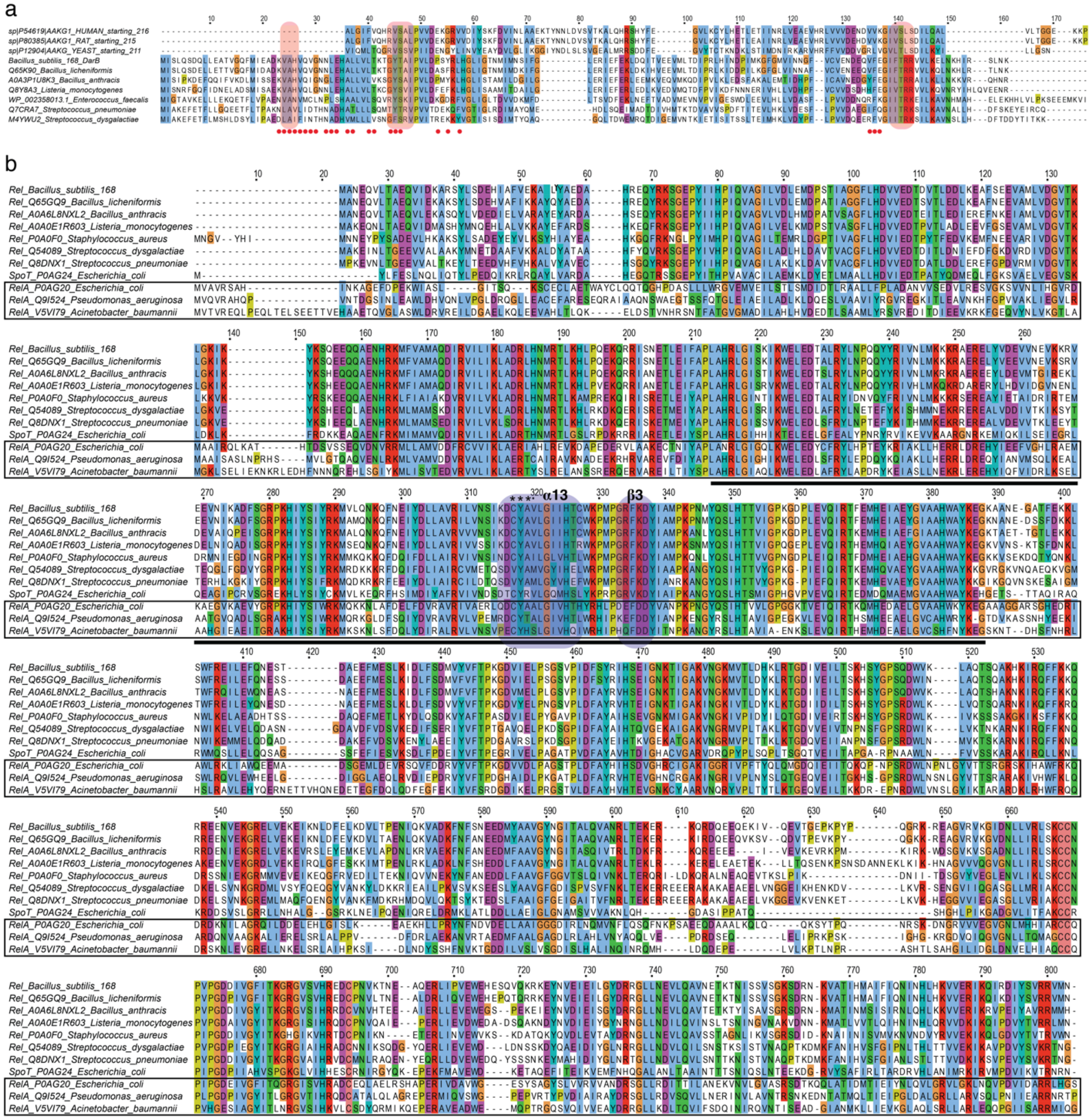

Fig. S3.

c-di-AMP-induced allosteric rearrangements of the DarB dimer preclude the binding to Rel$_{Bs}$. (**a**) Analysis of the structural effect of the binding of c-di-AMP on the symmetry of the DarB dimer. Molecular details of the c-di-AMP interactions are shown in the panel to the right. (**b**) Details of the movement of α1 that underlies the allosteric rearrangement induced by c-di-AMP. The conformation of DarB in the complex with Rel$_{Bs}^{NTD}$ is shown in pink and in the complex with c-di-AMP is shown in dark magenta, Rel$_{Bs}^{NTD}$ SYNTH is in yellow. (**c**) Deviation of the DarB dimers in different states *vs* the ideal symmetry.

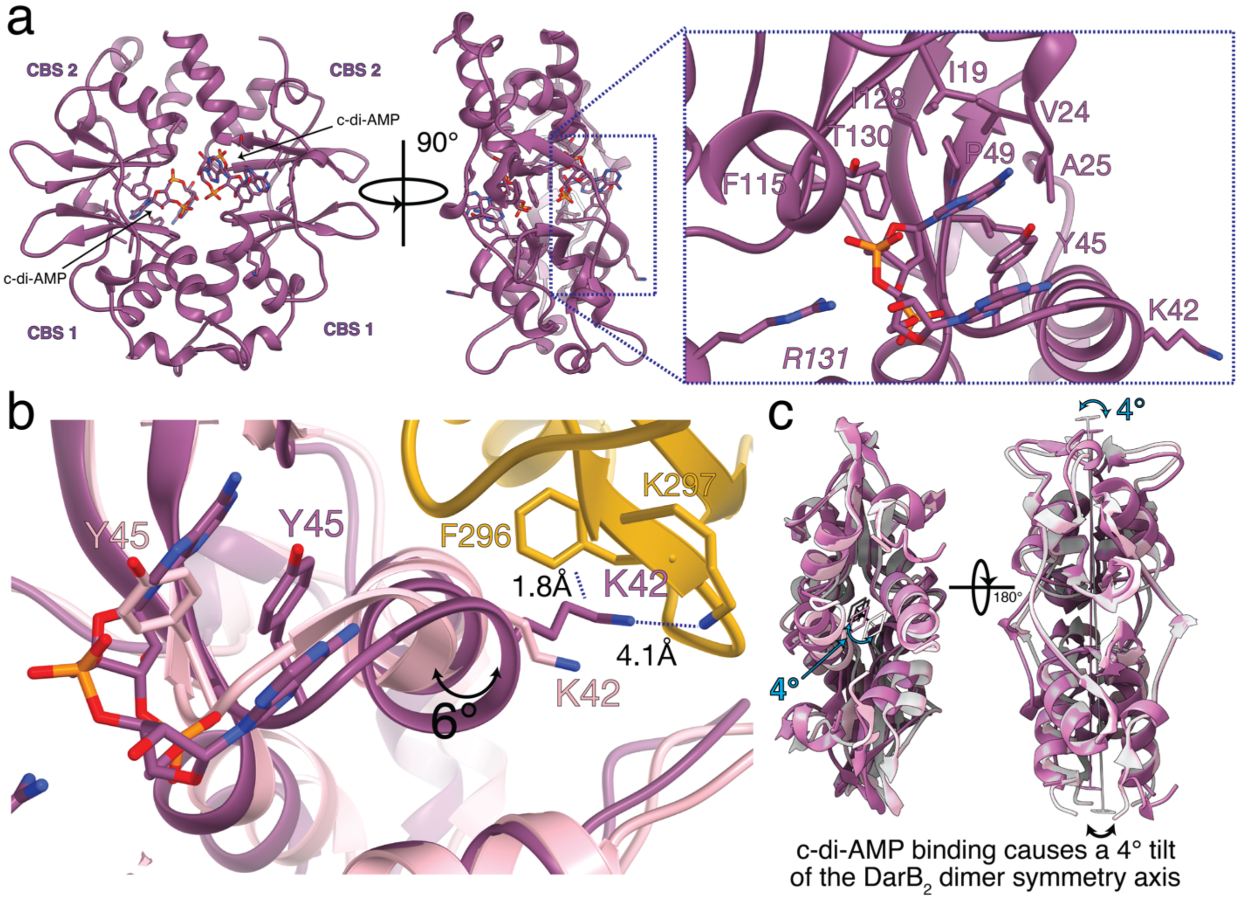

Fig. S4.

Conservation of the amino acid residues altered in DarB-suppressing mutants. (**a**) Superposition of *L. monocytogenes* CBP (PDB ID 6xnv), coloured in blue on *B. subtilis* DarB (this work) coloured according to Fig. 4f. (**b**) Detail of the conservation of *B. subtilis* DarB α1 residues (and their *L. monocytogenes* counterpart) involved in the binding to Rel. The residues that when substituted in *L. monocytogenes* CBP suppressed the interaction with Rel (Peterson *et al.* 2020 *(30)*) are shown in bold.

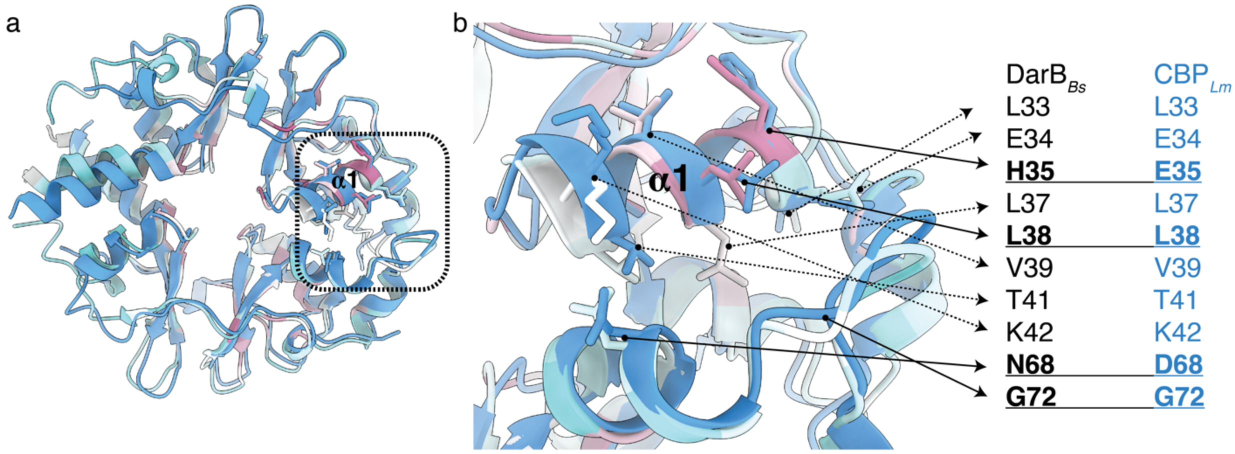

Fig. S5.

Different conformations of *B. subtilis* Rel$_{Bs}$. Comparison of *B. subtilis* Rel$_{Bs}^{NTD}$ in the DarB$_2$Rel$_{Bs}^{NTD}$$_2$ complex (coloured as in **Fig. 2** with the HD domain in blue and the SYNTH domain in yellow) with that of the crystallisation dimer of *B. subtilis* Rel$_{Bs}^{\Delta ZFD \Delta RRM}$ observed in 6YXA (coloured in dark green and light green). The incompatibility between these states becomes apparent from the comparison, with the TGS and helical domains of the crystallisation dimer protruding through DarB (in pink and lilac).

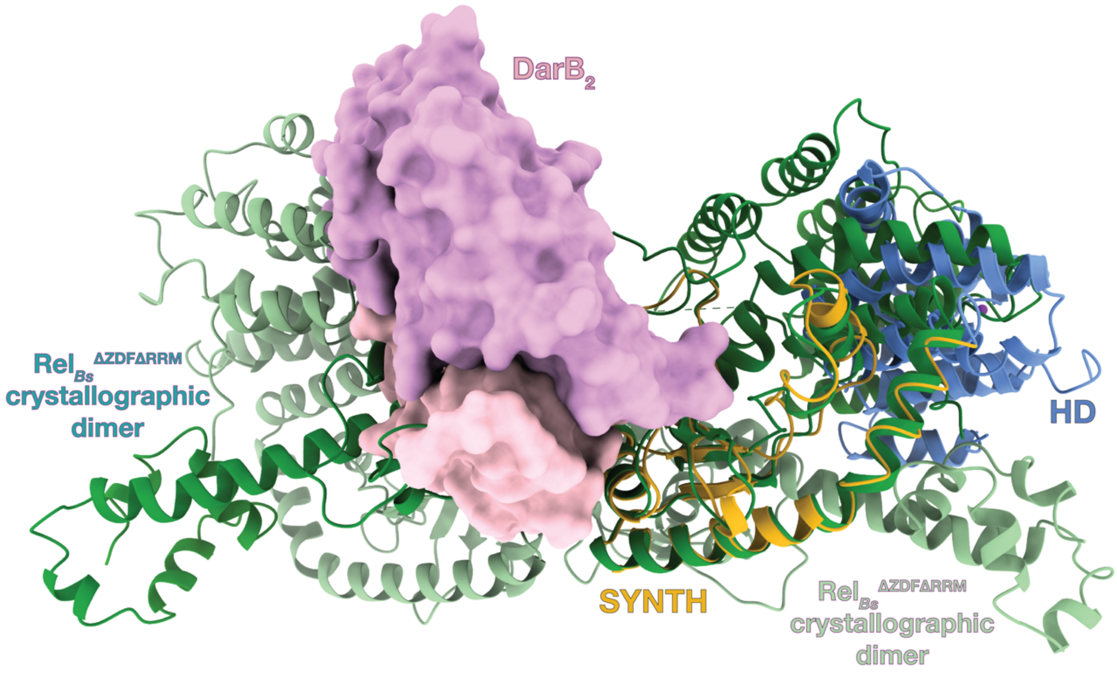

Supplementary Tables

Table S1.

Supplementary Table S1. Isothermal Titration Calorimetry (ITC) parameters. Experimentally determined binding thermodynamic parameters resulting from ITC measurements.

**Titrations**	***K_D*** **(µM)**	***ΔH*** **(kcal/mol)**	***-TΔS*** **(kcal/mol)**	***ΔG*** **(kcal/mol)**
c-di-AMP into DarB	0.0457	-11.1	1.3	-9.8
DarB into Rel_{Bs}^{NTD}	1.4	5.3	-13.1	-7.8
DarB-c-di-AMP into Rel_{Bs}^{NTD}	–	–	–	–
DarB into Rel_{Bs}^{NTD}_Y279A	4.0	3.3	-10.5	-7.2
DarB into Rel_{Bs}^{NTD}_K290G	45.1	1.1	-6.9	-5.8
DarB_E34R into Rel_{Bs}^{NTD}	10.6	3.2	-9.9	-6.7
DarB_E74G R75G into Rel_{Bs}^{NTD}	8.9	3.4	-10.3	-6.9
APCPP into Rel_{Bs}^{NTD}	–	–	–	–
APCPP into Rel_{Bs}^{NTD}-DarB	115.4	-2.2	-3.1	-5.3
pppGpp into Rel_{Bs}^{NTD}-DarB	8.2	-6.9	0.1	-6.8

Table S2.

Supplementary Table S2. X-ray data collection and processing.

The $CC_{1/2}$ criterion was used to determine the resolution range. Values for the outer shell are given in parentheses.

**Sample**	**DarB-c-di-AMP complex**	**Rel$_{Bs}$^{NTD}-DarB complex**
Diffraction source	Soleil PX2	Soleil PX2
Wavelength (Å)	0.9801	0.9801
Temperature (K)	100	100
Detector	Eiger-X 16M	Eiger-X 16M
Crystal-detector distance (mm)	200.0	250.1
Rotation range per image (°)	0.1	0.1
Exposure time per image (s)	0.004	0.004
Space group	$P2_12_12_1$	$P2_12_12_1$
a, *b*, *c* (Å)	105.6 42.3 65.0	94.4, 98.1, 126.8
α, β, γ (°)	90.0, 90.0, 90.0	90.0, 90.0, 90.0
Mosaicity (°)	0.20	0.20
Resolution range (Å)	52.81 – 1.52 (1.57 – 1.52)	77.60 – 2.97 (3.08 – 2.97)
Total N°. of reflections	294714 (3410)	452386 (23845)
N°. of unique reflections	40317 (998)	22489, (1023)
Completeness (ellipsoidal %)	88.0 (45.0)	095.0 (76.9)
Redundancy	7.3 (3.4)	20.1 (23.3)
$\langle I/\sigma(I)\rangle$	19.2 (2.3)	11.1 (1.1)
$CC_{1/2}$	0.999 (0.826)	0.998 (0.387)
R_{pim}	0.021 (0.251)	0.061 (0.851)
Overall *B* factor / Wilson plot (Å^2)	16.6	81.4
R-factor (%)	17.4	26.4
R_{free}-factor (%)	19.4	31.9
Ramachandran profile (%)		
Core	98.9	97.6
Allowed	1.1	2.4
Outliers	0.0	0.0
R.m.s. deviations		
Bond lengths (Å)	0.006	0.003
Bond angles (°)	0.86	0.63
Number of atoms	2550	7223
Macromolecules	2191	7179
Solvent	271	42
Other	88	2
B-factors (Å2)		
All atoms	22.7	88.2
Macromolecules	22.0	88.2
Solvent atoms	30.9	76.5
Other atoms	15.6	107.4
PDB ID	8AD6	8ACU

Table S3.

Supplementary Table S3. Strains and plasmids used in this study.

Plasmids	Description	Reference
pET24d-His10-SUMO kmR	Expression vector for SUMO-tagged protein	Laboratory stock
pET24d-His10-SUMO-DarB kmR (VHP731)	Expression vector for SUMO-tagged wt *B. subtilis* DarB	This study
pET24d-His10-SUMO-E36EDarB kmR (VHP1224)	Expression vector for SUMO-tagged E36R-subsituted *B. subtilis* DarB	This study
pET24d-His10-SUMO-rel kmR (VHP186)	Expression vector for SUMO-tagged *B. subtilis* Rel	(Takada *et al.*, 2020a) *(34)*
Strains		
E. coli BL21 DE3	B F^{-} ompT *gal dcm lon hsdSB*(*rB*$^{-}$*mB*$^{-}$) λ(DE3 [*lacI lacUV5-T7p07 ind1 sam7 nin5*]) [*malB*$^{+}$]$_{K\text{-}12}$(λ^{S})	Laboratory stock

Supplementary Data 1

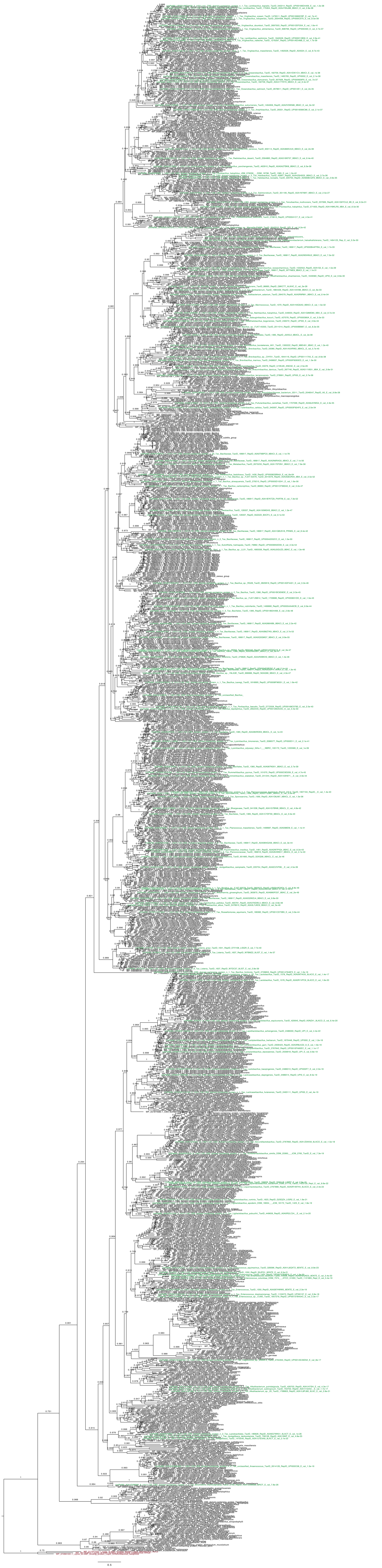

Supplementary Data Legend:

Supplementary Data 1. CBS domain phylogenetic tree. DarB proteins used in the ConSurf analysis are coloured in green.

Supplementary Data 2. ID of the Rel sequences that were used in the ConSurf analysis.

Supplementary Data 3. ID of the DarB sequences that were used in the ConSurf analysis.
